# Incidence of human brucellosis in a rural area in Western Greece after the implementation of a vaccination programme against animal brucellosis

**DOI:** 10.1186/1471-2458-8-241

**Published:** 2008-07-17

**Authors:** Eleni Jelastopulu, Christos Bikas, Chrysanthos Petropoulos, Michalis Leotsinidis

**Affiliations:** 1 Department of Public Health, Medical School, University of Patras, Patras, Greece; 2 Health Centre of Erymanthia, Erymathia, Greece

## Abstract

**Background:**

Brucellosis continues to be an important source of morbidity in several countries, particularly among agricultural and pastoral populations. The purpose of this study was to examine if there is an effect on the incidence of human brucellosis after the implementation of an animal brucellosis control programme.

**Methods:**

The study was conducted in the Municipality of Tritaia in the Prefecture of Achaia in Western Greece during the periods 1997–1998 and 2000–2002. Health education efforts were made during 1997–1998 to make the public take preventive measures. In the time period from January 1999 to August 2002 a vaccination programme against animal brucellosis was realised in the specific region. The vaccine used was the *B. melitensis *Rev-1 administered by the conjuctival route. Comparisons were performed between the incidence rates of the two studied periods.

**Results:**

There was a great fall in the incidence rate between 1997–1998 (10.3 per 1,000 population) and the period 2000–2002 after the vaccination (0.3 per 1,000 population). The considerable decrease of the human incidence rate is also observed in the period 2000–2002 among persons whose herds were not as yet vaccinated (1.4 vs. 10.3 per 1,000 population), indicating a possible role of health education in the decline of human brucellosis.

**Conclusion:**

The study reveals a statistically significant decline in the incidence of human brucellosis after the vaccination programme and underlines the importance of an ongoing control of animal brucellosis in the prevention of human brucellosis. The reduction of human brucellosis can be best achieved by a combination of health education and mass animal vaccination.

## Background

Brucellosis, a zoonotic illness caused by different species of *Brucella*, remains a serious problem of public health for countries around the Mediterranean Sea, Arabic Peninsula and some countries in Latin America and Asia [1]. These pathogenic bacteria can not only infect sheep, goats, cattle, pigs and camels, but humans as well. *B. melitensis *(sheep and goats) is the most important causative agent for human brucellosis followed by *B. abortus *(cattle), *B. suis *(pigs) and *B. canis *(dogs).

Brucellosis is endemic in Greece; however incidence, where reported, is likely to underestimate the true disease burden. *B. melitensis *is the most common pathogen in Greece [2]. In the period from 1993 to 2000 there had been an increase in the reported cases from 112 to 548 cases (1.1 to 5.0 per 100,000), whereas after the year 2000 a remarkable decrease in the number of reported cases was observed (2.2 per 100,000 in 2003) [3]. However, recent studies reported an annual incidence of 17.3 – 1,110 per 100,000 population for human brucellosis in certain rural areas of Greece [4-6].

Control strategies available to prevent human brucellosis are pasteurization of milk, livestock vaccination and elimination of infected animals. In animal populations, control of infection can be based on different strategies the selection of which depends on numerous factors. Mass vaccination accompanied by a strict surveillance scheme is a first step to reduce the number of infected animals and hence the infection pressure in regions where the incidence rate of animal brucellosis is high [7]. At a low level of infection a test-and-slaughter programme can be applied in order to attain brucellosis free flocks and zones [8]. The most commonly used vaccines are *B. melitensis *Rev-1 and *B. abortus *S19 vaccines. *B. abortus *RB51 vaccine is used in some countries on a small scale [9]. The *B. melitensis *Rev-1 strain is currently considered as the best vaccine available for the control of ovine and caprine brucellosis, especially when used at the standard dose by the conjunctival route [10].

The Greek Ministry of Agriculture, in a co-operation with the European Union, applied a countrywide vaccination programme in order to reduce the incidence of animal brucellosis [11]. Since the incidence of human brucellosis correlates with the extent of an animal vaccination programme, it is very interesting to investigate the degree of the decline in the incidence of human brucellosis in those areas where vaccination was applied. Therefore, the main objective of this study was to investigate the above effect of animal vaccination on human brucellosis incidence rate and additionally to investigate the impact of health education on the disease incidence.

## Methods

The study was conducted in the Municipality of Tritaia in the Prefecture of Achaia in Western Greece during the periods 1997–1998 (first period) and again during 2000–2002 (second period). The mean population of the studied area in this period was 6,121 according to the census of the year 2001. The population of the region is mainly pastoral (stock-breeders whose main source of income comes from selling dairy products, meat, eggs etc.) and the incidence of both animal and human brucellosis was very high until the year 1998 in relation to the national incidence of the disease (1.1 per 100,000 – 4.2 per 100,000 from 1993–1998 for human brucellosis).

During the first period (1997–1998) a case-control study was conducted, in order to investigate risk factors as well as a simultaneous incidence rate study [4]. This study comprised 123 patients who were examined in the local Health Centre for diagnosis or follow-up as well as 274 controls from the same region matched for age and gender. Diagnosis of the human brucellosis was established by the positive Standard Agglutination Test (SAT) where a titer of ≥1/160 or increasing in time in addition to defined clinical symptoms and signs such as fever, fatigue, arthralgia etc. was considered positive. In all cases, we considered a diagnosis to be a long term process of more than two weeks, during which clinical examination is performed several times as well as the Standard Agglutination Test. No brucella cultivation was performed but successful specific treatment was also used as a diagnostic criterion of the disease. Given that the follow up (including the prescription of drugs) of all cases was performed exclusively by the local medical staff, it can be concluded that this study comprised almost all patients in the region during the studied periods.

All participants in this study received home visits, during which health education talks were organised to all family members of the participants. During these talks contamination conditions were described and analyzed in detail. For instance, emphasis was made on milk pasteurization, use of individual protection devices during delivery, milking and slaughtering (gloves, mask). All the above elements were revealed to be important contamination factors as shown in the previous case-control study [4]. It should be added that public health education efforts (radio broadcasts, open public meetings, etc.) were performed by the local Health Centre's medical staff. The economic benefits of disease control for both animal and human brucellosis was particularly stressed to the whole population of the studied area.

In the time period between September 1999 and August 2002 an animal vaccination campaign was conducted by the Field Veterinary Service (FVS) of the Greek Ministry of Agriculture in addition to other eradication efforts such as health education of the population and animal movement control which included an official veterinary certificate by the FVS (Table 1). This certificate contained detailed information on the health status of the flock, the purpose of the movement and the final destination, especially during transhumance period. The final objective was to establish that flocks grazing in common pastures must have the same health status.

**Table 1:** Annual animal vaccination coverage in Tritaia in the years 1999–2002

	Herds			Animals		
Year	N	%	Cum %	N	%	Cum %

1999	24	3.5	3.5	15269	17.2	17.2
2000	156	22.8	26.3	22097	24.9	42.1
2001	476	69.7	96.0	48192	54.4	96.5
2002	20	2.9	98.9	2447	2.8	99.3
Unvaccinated	7	1.0	100	634	0.7	100
Total	683	100		88639	100	

The vaccination programme included the vaccination of all non-pregnant female sheep and goats aged over three months. The detailed descriptions of vaccination methods are found in the annual reports of the Food and Veterinary Office (FVO) of the European Commission [12]. The vaccine used was the *B. melitensis *Rev-1 (since *B. melitensis *is the main and most frequent pathogenic species in Greece) and was administered by the conjunctival route. Exclusively veterinary medical officers performed all vaccinations while health assistants immobilized the animal. Each dosage contained 1 × 10^9 ^CFU. The vaccination programme included almost all herds of the investigated region. Being more specific, at the end of the campaign, 676 out of 683(99%) herds were vaccinated, accounting for 88,005 of the 88,639 (99.3%) sheep and goats. The small number of dropouts was due to herds temporarily moving outside the study area and consequently, escaping the vaccination.

In the second period (2000–2002) of the study only an incidence rate study was realized in order to investigate the incidence of human brucellosis in persons whose herds were vaccinated and in persons whose herds were not yet vaccinated.

Comparisons were performed between the incidence rates of the two studied periods, i.e. before the vaccination campaign (1997–1998) and during the vaccination programme (2000–2002), separating those whose herds were vaccinated and those whose herds were not yet vaccinated.

Statistical analysis was performed by SPSS v. 15 statistical package (SPSS Inc. USA). Kolmogorov-Smirnov-test was applied in order to compare age distribution of cases before and after the vaccination campaign so that age deviations could be linked to variations in contamination conditions. Poisson regression was employed to assess the incidence rate ratios between the different time periods. The structure of the model was $m_{i,j}=N_{i,j}*e^{\mu +\alpha_{i}+\beta_{j}}$ where m_*i*, *j *_denotes the incidence rate ratio for i^th ^age group (i = 1,2...8), and j^th ^time period (j = 1,2), *N*_*i*, *j *_the corresponding estimated population at risk, *α*_i _represents the effect for i^th ^age group and *β*_j _represents the j^th ^time period effect.

The Plenary meeting of the School of Medicine approved the investigation, which was conducted by the staff of the Department of Public Health of University of Patras and the staff of the Health Centre of Erymanthia. Verbal consent was obtained from all study subjects.

## Results

Table 2 presents the annual incidence rates of human brucellosis per 1,000 population in the time period of 1997–1998 (before implementation of the vaccination programme) and 2000–2002 when vaccination programme was running, separating persons whose herds were already vaccinated and persons whose herds were not yet vaccinated.

**Table 2:** Annual incidence rates of human brucellosis per 1000 population in the study area

Period 1997–1998*	1997	1998		Mean incidence
	
	13.2 (79)**	7.3 (44)		10.3 (123)
Period 2000–2002***	2000	2001	2002	Mean incidence
	
before vaccination	1.1 (7)	2.5 (15)	0.5 (3)	1.4 (25)
after vaccination	0.0 (0)	0.2 (1)	0.7 (4)	0.3 (5)
total	1.1 (7)	2.7 (16)	1.2 (7)	1.7 (30)

* mean population: 5996

** in brackets, number of cases

*** mean population: 6121

During the second period (2000–2002) a statistically significant reduction of the incidence rate was observed, mainly between the period 1997–1998 (i.e. before vaccination – 10.3 per 1,000 population) and the period 2000–2002 after vaccination (0.3 per 1,000 population, p < 0.001). The reduction in the incidence has been already seen in 1998 (7.3 vs 13.2 per 1000), one year after health education was started. Statistically significant decrease in the human incidence rate is also observed in the period 2000–2002 even in persons, whose herds were not as yet vaccinated (1.4 vs. 10.3 per 1,000 population, p < 0.001). A further statistical significant reduction (0.3 vs 1.4 per 1000 population, 5 times lower) was achieved in persons whose herds were vaccinated, a fact that demonstrates the considerable effectiveness of the animal vaccination in the decline of human brucellosis [13] (see Tables 2 and 3).

**Table 3:** Incidence rate ratios of human brucellosis between periods 1997–1998 and 2000–2002

Period	Incidence rate ratio*	95% C.I.
2000–2002 vs 1997–1998	0.15	0.10 – 0.23
2000–2002 (bv**) vs 1997–1998	0.13	0.09 – 0.20
2000–2002 (av***) vs 2000–2002 (bv**)	0.20	0.08 – 0.52

* Poisson regression

** before vaccination

*** after vaccination

It is notable, as indicated in Table 4, that the age distribution of cases in 1997–1998 and 2000–2002 is quite identical and therefore the influences, which decrease the incidence of the disease, appear to be spread homogenously in the population.

**Table 4:** Age contribution of cases in the periods 1997–98 and 2000–02 (both sexes)

	1997–1998		2000–2002	
Age groups	Cumulative frequency	Cumulative percent (%)	Cumulative frequency	Cumulative percent (%)

0–9	6	5	1	3
0–19	17	14	3	10
0–29	35	28	7	23
0–39	49	40	9	30
0–49	67	54	17	57
0–59	84	68	21	70
0–69	105	85	25	83
0–80	123	100	30	100
p >0.05 (Kolmogorov-Smirnov test)				

* Poisson regression ** before vaccination *** after vaccination

## Discussion

In many countries it has been observed that there is a very close positive correlation between incidence of brucellosis in animals and incidence of the disease in humans [14]. Therefore, animal vaccination should considerably influence the human incidence of the disease. Furthermore, studies of risk factors have concluded that several behavioural items (dairy products consumption, animal delivery practices) play a very important role in the spread of the disease [4]. The respective role of vaccination and health education is a very important subject of future studies. In this study we have observed that there is a substantial reduction in the annual incidence rate between the two studied periods in persons whose herds were not yet vaccinated. This reduction could be attributed partially to the health education efforts and the changes in hazardous habits (pasteurization of dairy products, using masks and gloves, especially during animal delivery etc.). Moreover, a favourable role plays the fact that in the period 2000–2002, a lot of herds were already and gradually vaccinated and therefore contamination conditions were somehow modified even in persons whose herds were not as yet vaccinated.

The fact that the incidence rate after the animal vaccination was decreased from 1.1 to 0.3 per 1,000 population indicates that reduction of human brucellosis may be realized, proved that animal vaccination is correctly and completely performed and health education is well established in the population. The small number of cases (five in the studied area) identified after vaccination campaign was probably due to the exposure to infected material, food or animals in that area or from other regions. In fact, although it is known that vaccine itself can be an infective agent to man, we have not identified any possible contamination of the cases by the vaccine itself given that none of them was involved in vaccination process [15].

These results are in agreement with the results of Minas A. et al. who has observed that there is a very close correlation between vaccination programmes and human incidence rate; and in cases where vaccination was very intensive, human incidence rate was declining significantly [16]. In contrast, whenever animal vaccination was interrupted, human incidence rate tended to increase considerably, especially in cases where no health education programmes were ever implemented [14,16].

Our results demonstrate that even when time period is not taken into consideration (as in period 2000–2002, when incidence rate declined from 1.4 to 0.3 per 1,000 population) the vaccination of one's herd appears to decrease considerably the probability of contamination of the family members.

Brucellosis has serious economic effects in the local population and can cause serious problems in the national agricultural economy as well. Since slaughtering is the only solution when an animal is infected it is obvious that a high incidence of animal brucellosis leads to great losses in breeder's income. In Mongolia, a well-designed survey was conducted recently in order to estimate the economic benefits from livestock vaccination for brucellosis, where the total costs for the vaccination (vaccines, veterinaries fees, transportations, ear tagging etc) were compared to the economic benefits after vaccination, such as avoidance of losses in animals and animal products, decline in the human disability to work, reduction of treatments cost etc [17]. The study demonstrated a high cost-effectiveness of the vaccination and a theoretical great net economic benefit for the farmers. Unfortunately, in Greece, no cost-effectiveness studies have been performed till now to support similar financial benefits from livestock vaccination.

## Conclusion

In this study we observed that a considerable reduction of incidence of the disease was achieved by combination of health education and animal vaccination. The incidence rate decreased from 13.2 per 1,000 population in 1997 to 0.7 per 1,000 population in 2002 after animal vaccination. It is obvious that the reduction of brucellosis in both animals and humans can be achieved with a mass animal vaccination in combination with health education, improving not only farmer's health but their economic situation as well.

## Competing interests

The authors declare that they have no competing interests.

## Authors' contributions

EJ had the original idea for the study, carried out the statistical analysis, formed the layout of the manuscript and co-wrote the final manuscript. CB developed the questionnaire, performed data interpretation, wrote the first draft of the manuscript. CP performed data collection and contributed to the preparation of the manuscript. ML carried out further statistical analysis and contributed to the final manuscript. All authors read and approved the final manuscript.

## Pre-publication history

The pre-publication history for this paper can be accessed here:


